# Topics of Public Interest

**Published:** 1908-09

**Authors:** 


### TOPICS OF PUBLIC INTEREST.

---

### The Antituberculosis Crusade at the County Fairs.

"Every thirty-six minutes there is a death from consumption in the State of New York," so read several charts of the tuberculosis exhibitions of the State Charities Aid Association, which are to be shown at thirty-six of the county fairs of this state during the coming fall. To many this is a bit of news at once surprising and startling. The great majority of people have no conception of the awful ravages of this disease, of that sorrow, suffering, misery, destitution and squalor that are left in the trail of the "Great White Plague," more profound still is the ignorance of the nature of the disease. Believing and regarding it as hereditary and incurable, humanity has from time immemorial suffered tuberculosis to rage unchecked, but now however, thanks to science, it has been known for some twenty years that this plague is not only not hereditary, but that it is curable in the early stages, and most important of all, that it is preventable, perhaps more so than any other germ disease.

To disseminate our present day knowledge of the means and methods for checking and securing a control over this scourge, an educational campaign was carried on in nine cities of this state. during the past year. But the people living in the country should get the message also, to them should be imparted our information about the disease, for though it is not generally known that in the small towns and rural districts of this state tuberculosis is almost as prevalent in proportion to the population, as it is in the large cities, still such is the case, however, as has been determined from an examination of the vital statistics of all the towns and villages of the state.

The county fair offer a splendid opportunity of reaching the people of the rural districts, and of bringing home to them the essential facts about this disease, its nature, its extent, how it spreads, how it may be cured and prevented. Over 400,000 people, it is expected, will hear the gospel of the antituberculosis crusade.

Each exhibit will be made up of maps, charts, diagrams, models of well lighted and ventilated factories, together with models of sweat shop work-rooms, and dark poorly ventilated bedrooms where the consumption germ finds an excellent breeding place. Pictures of dispensaries, hospitals; and sanatoria where consumptives are treated, radiographs and photographs of healthy and diseased lungs will be shown. Scores of short terse pointed texts will be displayed about the exhibit.

Nothing gruesome or objectionable has been allowed to creep into the exhibits, so no one need stay away on that account. The tone throughout is optimistic for it has been felt that in a great, tremendous campaign like this, the greatest perhaps that ever has or ever will be carried on by preventive medicine, optimism is a prime requisite.

---

UNUSUAL CARDIAC MURMURS.—Richard Ellis, of New York, (*Medical Record,* July 25, 1908,) gives twenty-one cases in healthy persons in whom there were cardiac murmurs, in many cases causing them to be rejected by life insurance companies, while no organic lesions of the heart could be discovered by the author. Several of these were double murmurs synchronous with respiration and caused in some way by the act.

